# Supplementary material for: Extracellular Vesicles May Predict Response to Atezolizumab Plus Bevacizumab in Patients with Advanced Hepatocellular Carcinoma
Source: Cancers (Basel). 2024 Oct 29;16(21):3651. doi: 10.3390/cancers16213651 (PMC11545167; doi:10.3390/cancers16213651)
Supplement: Supplementary file 1 [file cancers-16-03651-s001.zip › cancers-3248490 - supplementary.pdf]

Supplementary Table S1: Correlation of tumor characteristics and longitudinal changes of vesicle characteristics with objective response. Data is shown as univariate Cox-regression analysis.

Abbreviations: ALBI-score, albumin-bilirubin-score; ALD, alcohol-related liver disease; BCLC, Barcelona Clinic Liver Cancer; CI, confidence interval; EHS, extrahepatic spread; HR, hazard ratio; MASH, metabolic dysfunction-associated steatohepatitis; MVI, macrovascular invasion; T0, therapy naive; T1, 3 weeks after therapy induction; T3, 9 weeks after therapy induction.

	P	HR	CI
**Vesicle size [nm]**			
Interval T0-T1	0.539	0.67	0.18-2.43
Interval T0-T3	0.084	0.26	0.10-1.20
Interval T1-T3	0.213	0.38	0.08-1.74
9 weeks after induction	0.040	0.18	0.03-0.93
**Vesicle concentration [particles/mL]**			
Interval T0-T1	0.364	2.00	0.45-8.94
Interval T0-T3	0.241	2.40	0.56-10.38
Interval T1-T3	0.213	0.38	0.08-1.74
**Zeta potential [mV]**			
Interval T0-T1	0.484	1.60	0.43-5.96
Interval T0-T3	0.222	0.39	0.08-1.77
Interval T1-T3	0.035	0.15	0.03-0.87
gender	0.684	1.46	0.236-8.90
Cirrhosis	0.611	0.65	0.12-3.49
Viral	0.199	2.38	0.64-8.88
ALD	0.645	0.75	0.22-2.59
MASH	0.947	1.08	0.10-11.36
BCLC	0.274	0.45	0.11-1.89
Child Pugh Score	0.909	0.91	0.19-4.33
ALBI- Score	0.324	1.91	0.53-6.94
MVI	0.041	0.25	0.07-0.94
EHS	0.872	0.90	0.25-3.24

Supplementary Table S2: Correlation of tumor characteristics and longitudinal changes of vesicle characteristics with duration of response. Data is shown as univariate Cox-regression analysis**.**

Abbreviations: ALBI-score, albumin-bilirubin-score; ALD, alcohol-related liver disease; BCLC, Barcelona Clinic Liver Cancer; CI, confidence interval; EHS, extrahepatic spread; HR, hazard ratio; MASH, metabolic dysfunction-associated steatohepatitis; MVI, macrovascular invasion; T0, therapy naive; T1, 3 weeks after therapy induction; T3, 9 weeks after therapy induction.

	P	HR	CI
**Vesicle size [nm]**			
Interval T0-T1	0.985	0.99	0.46-2.16
Interval T0-T3	0.051	2.62	0.90-6.90
Interval T1-T3	0.188	2.05	0.71-5.96
9 weeks after induction	0.214	0.38	0.08-1.74
**Vesicle concentration [particles/mL]**			
Interval T0-T1	0.682	0.83	0.342-2.02
Interval T0-T3	0.588	0.77	0.30-1.97
Interval T1-T3	0.580	0.74	0.25-2.16
**Zeta potential [mV]**			
Interval T0-T1	0.594	1.24	0.57-2.70
Interval T0-T3	0.683	1.24	0.44-3.54
Interval T1-T3	0.138	0.43	0.14-1.32
gender	0.009	0.20	0.06-0.67
Cirrhosis	0.364	1.49	0.63-3.49
Viral	0.149	0.59	0.29-1.21
ALD	0.125	1.87	0.84-4.15
MASH	0.143	2.52	0.73-8.65
BCLC	0.178	0.59	0.28-1.27
Child Pugh Score	0.276	1.27	0.83-1.93
ALBI- Score	0.032	2.17	1.07-4.40
MVI	0.089	0.52	0.25-1.10
EHS	0.356	0.71	0.34-1.48
